# FACET – a “Flexible Artifact Correction and Evaluation Toolbox” for concurrently recorded EEG/fMRI data

**DOI:** 10.1186/1471-2202-14-138

**Published:** 2013-11-09

**Authors:** Johann Glaser, Roland Beisteiner, Herbert Bauer, Florian Ph.S Fischmeister

**Affiliations:** 1 Biological Psychology Unit, Faculty of Psychology, University of Vienna, Liebiggasse 5, A-1010 Vienna, Austria; 2 High Field MR Centre of Excellence, Medical University of Vienna, Lazarettgasse 14, A-1090 Vienna, Austria; 3 Study Group Clinical fMRI, Department of Neurology, Medical University of Vienna, Währinger Gürtel 18-20, A-1090 Vienna, Austria

## Abstract

**Background:**

In concurrent EEG/fMRI recordings, EEG data are impaired by the fMRI gradient artifacts which exceed the EEG signal by several orders of magnitude. While several algorithms exist to correct the EEG data, these algorithms lack the flexibility to either leave out or add new steps. The here presented open-source MATLAB toolbox FACET is a modular toolbox for the fast and flexible correction and evaluation of imaging artifacts from concurrently recorded EEG datasets. It consists of an Analysis, a Correction and an Evaluation framework allowing the user to choose from different artifact correction methods with various pre- and post-processing steps to form flexible combinations. The quality of the chosen correction approach can then be evaluated and compared to different settings.

**Results:**

FACET was evaluated on a dataset provided with the FMRIB plugin for EEGLAB using two different correction approaches: Averaged Artifact Subtraction (AAS, Allen et al., NeuroImage 12(2):230–239, 2000) and the FMRI Artifact Slice Template Removal (FASTR, Niazy et al., NeuroImage 28(3):720–737, 2005). Evaluation of the obtained results were compared to the FASTR algorithm implemented in the EEGLAB plugin FMRIB. No differences were found between the FACET implementation of FASTR and the original algorithm across all gradient artifact relevant performance indices.

**Conclusion:**

The FACET toolbox not only provides facilities for all three modalities: data analysis, artifact correction as well as evaluation and documentation of the results but it also offers an easily extendable framework for development and evaluation of new approaches.

## Background

Concurrent acquisition of electroencephalogram (EEG) and functional magnetic resonance imaging (fMRI) allows combining the strength of both methods and completing the fragmentary information provided by either one of them [1]. Since the first publication of the simultaneous recording of EEG and BOLD contrasted MRI in 1993 [2] great efforts have been made to turn this powerful tool into a standardized technique. Thus combined acquisition of EEG and fMRI has turned into an essential tool not only to answer basic neuroscientific research questions, e.g. related to tonic alertness [3], resting-state connectiviy [4] or changes of sleep patterns [5,6], but also in clinical applications, in particular to localize the sources of ictal and interictal epileptic neural activity and networks [7,8].

These advantages of extended insights into brain activity come with drawbacks like mutual interference between the acquisition equipment. While artifacts in MRI images and fMRI data induced by the EEG equipment are minimal or even do not exist [9], scanner-induced artifacts in EEG recordings are quite substantial and their elimination is challenging. Basically, these artifacts can be divided into two groups: (1) artifacts related to the cardiac pulse, which causes movements of the electrodes within the static magnetic field (*B*_0_) of the MR-scanner [10] and (2) artifacts produced by the fast switching of the magnetic field gradients [11]. However, although well understood, gradient artifacts exceed the amplitude of the recorded EEG signals by several orders of magnitudes. Additionally, the raw artifact amplitude strongly depends on the change rate of the magnetic gradient field (which itself depends on the utilized fMRI sequence), the EEG electrode wire paths (loop area, orientation in the magnetic field), the EEG channel and the position of the head in the scanner [11]. The removal of this artifact is therefore a non-trivial task even when using optimized registration setups [12].

Several algorithms for the correction of these artifacts (see [13-15] for reviews) have been proposed, ranging from average artifact subtraction [16] to multi-step methods including various pre- and post-processing steps [17-19]. Yet, these algorithms commonly address only certain specific problem. For example, the problem of unsynchronized clocks of the EEG recording system and the MR scanner was tackled by both, the interpolation–template–alignment–subtraction (ITAS) algorithm [14] which aligns every EEG epoch to maximize its cross-correlation with a reference epoch following an up-sampling of the data or by the retrospective synchronization algorithm [20]. Slow drifts or subtle subject movements are commonly tackled using block-wise generation of templates [16], weighted templates [14] or using only every second slice to reduce possible correlations between them [17]. Still problematic are abrupt changes of the artifact signal, i.e. subject movements of 1 mm have been shown to already impair the results. In order to rectify this problem a realignment parameter informed algorithm [18] can be used which introduces the use of fMRI realignment parameters calculated from the image data.

Most of these described correction algorithms are freely available to the scientific community, while others are either not available or my even be proprietary. Available implementations are tailored to correct specific aspects of MR-related artifacts, implementing the individual correction steps in a fixed order and thus do not allow for a flexible configuration or combination of different correction approaches. However, a more flexible approach is of tremendous importance, since, depending on the EEG dataset not every corrective step is necessarily required and may even have adverse effects on the results. On the other hand, additional steps or innovative combinations of present steps can contribute to an improvement of the results. For example, the template subtraction can be performed twice with different methods for the template generation.

Here we present a new toolbox called FACET (“Flexible Artifact Correction and Evaluation Toolbox”) aiming to combine different correction steps in a flexible and extensible way using a single framework. Dedicated tools for the analysis of the EEG input data as well as methods to correct faulty trigger recordings are implemented as well. The toolbox is complemented by a dedicated framework for the evaluation of artifact correction algorithms using selected performance indicators.

Throughout the remainder of this manuscript we first describe the implementation of the toolbox followed by an in depth description of the currently implemented set of utilities, i.e. the most popular correction algorithms. To provide the user with insights into setup and execution of implemented algorithms, the setup demonstrating the functionality of the toolbox will be explained using short application examples throughout the manuscript. Finally, the functionality of FACET will be demonstrated on a dataset supplied with the FMRIB plugin by Niazy et al. [17] using the popular algorithm by Allen [16] and Niazy [17]. All scripts to demonstrate the functionality of FACET are provided with the FACET package as well as in the appendix (Additional files 1 and 2).

## Implementation

FACET – the artifact correction and evaluation toolbox – consists of an ANALYSIS, a CORRECTION and an EVALUATION framework and relies on the EEGLAB data structure [21]. EEGLAB^a^ is a widely used and extensible open-source EEG processing toolkit program for Matlab. As a starting point the FASTR algorithm [17] and the FARM algorithm [19] were used. While FASTR is available as a plugin^b^ for EEGLAB, the source of FARM is not provided by the authors.

The ANALYSIS framework provides information on the EEG data at hand, useful for the further setup of the correction procedure. Within the CORRECTION framework, a selection of various algorithms for correcting imaging induced artifacts are provided: e.g. average artifact subtraction (AAS) [16], PCA based [17] or realignment-parameter informed [18] and adaptive template approaches [19]. These approaches can be combined with different pre- and post-processing steps e.g. high- and low-pass filters, volume onset detection [19], sub-sample alignment [19], principle component analysis [17] and adaptive noise canceling [16]. The EVALUATION framework of this toolbox allows to assess the quality of the chosen correction approach using a wealth of parameters to compare different settings. Additionally FACET offers functions to automatically generate LATE X code with tables and diagrams (using Ti*k*Z and PGFPlots) for direct inclusion in the user’s documentation.

FACET is implemented in the object-oriented programming paradigm. All classes are put together in the Matlab package FACET (note: it has the same name as the class of the correction toolbox). The whole package will be available at https://github.com/hansiglaser/facet under the terms of the GNU General Public License (GPL) to provide powerful collaboration, review, and code management. The archive includes documentation, examples, and all scripts used for the evaluation examples in this manuscript.

### Software design

**Figure 1:**
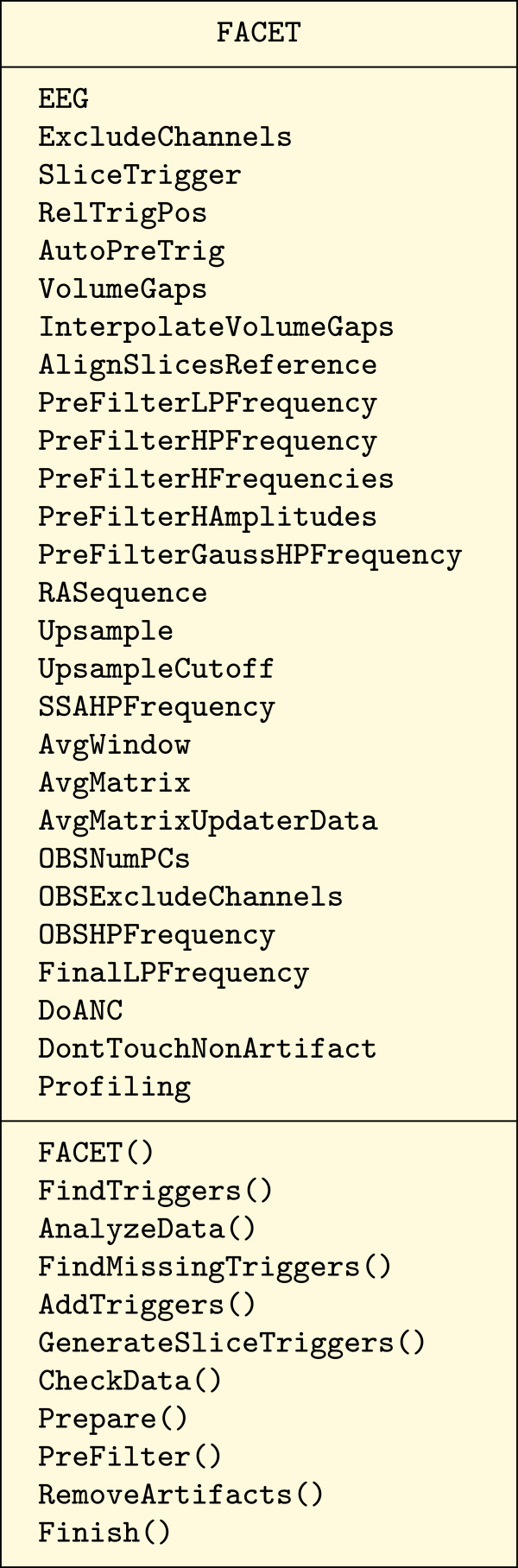
**Class diagram of the **FACET **base class of the artifact correction toolbox.** Only the public fields and methods are shown and the method parameters and return values are omitted.

Throughout the development of the FACET toolbox, no advantageous points were identified to split the data and/or functionality into multiple classes. Therefore, a single class “FACET” was created to hold all parameters and data. Its methods operate on this data to perform e.g., the artifact correction. Besides the data encapsulation, no other object oriented concepts like inheritance and polymorphism are used. Figure 1 shows the Unified Modeling Language (UML) class diagram for the correction framework. Public member fields for parameters and the EEG data are placed in the class. These have to be set by the user after instantiation of the class.

Keeping the EEG data inside the object as a member variable also improves memory management. In contrast, the FASTR plugin [17] uses functions and hands over the EEG data as function parameter and return value. In this case, Matlab creates a copy of the data every time. In the object-oriented approach, all methods work on the object member variables, therefore no copy is performed. This leads to reduced memory requirements (approximately by a factor of 3) and faster execution.

Several public methods are provided to analyze the data and remove the artifact. First of all, the constructor FACET() creates a new instance of the class. Other methods (see below) are used to identify (and optionally correct) trigger events and to print an analysis of the EEG data. Finally, the core algorithm is performed by executing its associated methods. The internal working is split into multiple private and protected methods. Additionally, events are issued at important points of execution to notify any interested programs. This allows data modification and printing of progress reports during the algorithm execution. The central class FACET silently performs the algorithm and does not print anything to the screen. The above mentioned events are used by the class FACET_Text, which is derived from FACET. It registers listeners to every event and prints a short message for every notification.

**Listing 1**Cleaning Example to demonstrate the concept of the CORRECTION framework.

Contrary to the graphical dialog user interface used in [17,18], FACET is fully configured with a configuration file (see for example Lst. 1). The user sets options and parameters for the algorithm before it is executed. This ensures reproducible results and allows the user to add comments for explanations and rationales for decisions. Using the object-oriented paradigm and employing Matlab itself as a scripting language enables the setup of the algorithm in exactly this way. The configuration values are assigned to public properties of the FACET object. Here the full power of Matlab is available to determine the value, either as a direct value, or calculated with user-defined formulas and even functions.

### The CORRECTION framework

**Figure 2:**
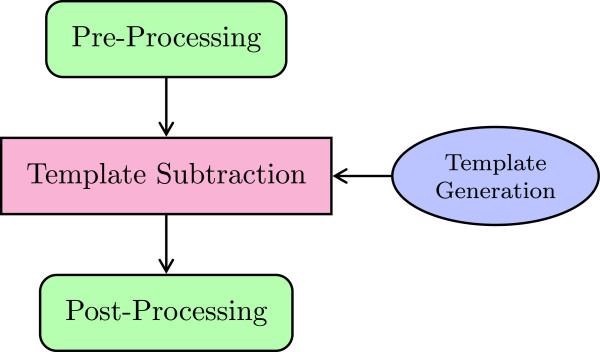
General approach of the artifact correction algorithms.

This section describes the CORRECTION framework. The generalized scheme for artifact reduction algorithms consists of several steps that can be summarized as pre-processing, template generation, and post-processing (see Figure 2). The pre-processing steps are introduced to avoid inaccuracies and thus residual artifacts in the following template subtraction step. The template generation step usually calculates the average of surrounding artifact epochs utilizing the fact that the artifact signal shape is a periodic function for the acquisition of every fMRI slice and/or volume. Depending on the exact algorithm, different epochs are selected for averaging. During post-processing, residual artifacts are commonly removed by applying principal component analysis or adaptive noise canceling and low-pass filtering.

#### Application Example 1: correction of EEG data

Before we describe the different methods and possibilities implemented in FACET a short application example will be provided to familiarize the reader with the general concept and with the individual steps needed. Overall, the toolbox provides several examples which will be used in excerpts throughout this work to demonstrate the features of the toolbox. The here shown example CleanEx1.m (short for “cleaning example number 1”) uses the FMRIB dataset and is given in extracts in Lst. 1. The full example with all definitions and extensive comments on most available functions can be found as a supplementary file (Additional file 1).

For easier execution of the example, it is wrapped in a Matlab function which accepts the raw EEG data in an EEGLAB dataset structure as an input parameter (line 4) and returns the cleaned data (line 38). This function is executed with the Matlab command line

where the variable EEG_FMRIB holds the FMRIB data set.

As first step, a FACET_Text object is instantiated (line 7) by executing the class constructor. The next step is to assign the raw EEG data to the public property EEG (line 11). Note that this property is internally implemented with a setter method. This automatically checks for the proper data format and extracts the number of channels and other values. Then, the function FindTriggers(EventType,TriggerOffset) (line 13) is used to find triggers with the label ’slice’ in the EEGLAB dataset and possibly correct their offset (here the offset is set to zero). In lines 17 and 20 two assignments of configuration values are shown. In the first case a direct value is assigned while the second case shows a formula using another configuration value. Actually, any Matlab construct can be used.

The first method called is AnalyzeData() in line 23. It is part of the ANALYSIS framework and prints information about the EEG data to the screen (see Lst. 2 for an exemplary output of the FMRIB dataset). Then, before the actual artifact correction algorithm is started, the data and the setup is checked for notable or problematic conditions using CheckData() in line 26. Execution of the actual artifact removal algorithm is invoked in lines 28–35. Prepare() derives some further internal variables from the setup configuration values. Then a filter (e.g. a high-pass) is applied to the EEG data using PreFilter(). This is part of the pre-processing step shown in Figure 2.

The main part of the correction algorithm is encapsulated in RemoveArtifacts(). It internally iterates over all EEG channels for which further pre-processing steps, template generation, template subtraction and post-processing are performed. The method Finish() simply stops the run-time counter and executes a final event notification. Finally, the cleaned EEG in the property EEG is returned to the caller (line 38).

#### Setup and preparation of data

Within FACET, several methods (c.f. Lst. 1 lines 13–26) are implemented to analyze and prepare the EEG, to check the setup, and to assign and correct MR related trigger information. All these steps aim to improve the following template subtraction step.

##### Analysis of EEG

Although users employing artifact correction algorithms are usually informed about the parameters of EEG and fMRI acquisition, methods are provided to analyse the EEG data to find problems, but also to find more detailed information.

To this end AnalyzeData() is used which characterizes the EEG dataset and displays information about it (see Lst. 2 for an exemplary output for the FMRIB dataset [17]). First all information stored within the EEGLAB dataset structure is printed, e.g. the number of samples, sampling rate, the number of channels including labels and a list of all event names that occur within the data together with their latency and frequency. Next, this function estimates the begin and end of the fMRI acquisition during the EEG recording – its duration as well as the duration of unimpaired EEG data before and after the fMRI acquisition are shown. If MR-acquisition related triggers are found, i.e. when the method FindTriggers (see below) was executed, a histogram of the distances between successive triggers is shown and used to warn if triggers are missing. It also checks if the number of triggers is enough for the duration of the fMRI acquisition. Here FACET automatically differentiates between volume acquisition and slice acquisition. The latter is assumed if the mean temporal distance between the triggers is below 1.0 s. For slice triggers, an analysis for volume gaps using a dedicated function to determine the gaps and to possibly interpolate, in a way similar to the FARM algorithm [19], can be performed and an estimate of total volume and slice count is printed. If volume triggers are assumed, slice periods are estimated by finding maxima in the auto-correlation function of one volume interval. This acquired information can later be used to convert the volume triggers to slice triggers.

**Listing 2** Exemplary output of AnalyzeData() for the FMRIB dataset.

##### Define and correct MR-Trigger

As a first step to prepare the execution of the artifact correction algorithm, the time instances of fMRI volume or slice acquisition have to be determined. In most cases the EEGLAB dataset will contain a list of trigger events, each characterized by a name and a latency value. FindTriggers() allows to filter these events specified by a given name and stores a list of all latency values in a member variable. The latency values will then be used by the template generation and subtraction steps. To correct for missing triggers, two methods are provided. First, users can scan the list of triggers for distances which are too large and can then insert missing volume or slice triggers automatically using FindMissingTriggers() (see Lst. 3). In the case of slice triggers, this method furthermore takes care to handle volume gaps correctly, i.e. larger intervals between the last slice trigger of one volume and the first trigger of the next volume are automatically detected and acknowledged.

**Listing 3** Example usage of FindMissingTriggers().

Since FindMissingTriggers() can only handle missing triggers between other triggers, but not e.g. before the first one to code for dummy-scans, additional methods are included in FACET allowing to manually add these missing events using AddTriggers() (c.f. Lst. 4).

**Listing 4** Example usage of AddTriggers().

Finally, as noted before, volume triggers can be converted to slice triggers. This routine (GenerateSliceTriggers()) can be used in all cases where the EEG data was recorded with triggers for the onset of every fMRI volume but without information on the slice onset. It creates a list of triggers from the number of slices per volume, the duration of every slice acquisition and the relative position of the slice acquisition within the volume acquisition.

##### Testing and finalizing of the setup

Finally, before the actual artifact correction algorithm is started, the data and the setup is checked for notable or problematic conditions. This is performed by the method CheckData(). The current implementation checks that EEG data is provided and that triggers were setup. Future extensions are planned to check filter properties for sensible values and certain interdependencies of the settings.

**Listing 5** Exemplary specification of the steps, which should be performed for artifact removal (from CleanEx1.m).

#### Prefiltering the data

**Figure 3:**
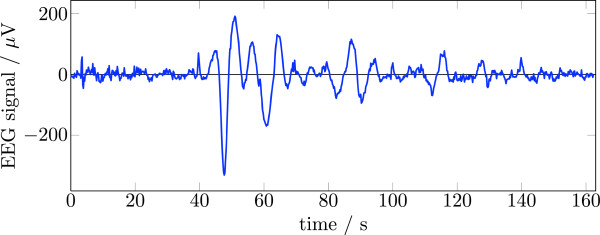
**Slow fluctuations of the baseline in the FMRIB dataset after correction with the FASTR algorithm **[17]** (note the time scale).**

Many EEG recordings collected during fMRI acquisition contain slow fluctuations of the baseline (see Figure 3). These are highly irregular and cause an unpredictable offset between the short slice epochs as well as a (nearly) linear trend of their baseline. These slow fluctuations have to be tackled with a high-pass filter with a very low cut-off frequency. Low-pass filter on the other side are sometimes used to remove the fMRI gradient artifacts (see e.g. [16]). The result often still shows large artifact amplitudes, because the base frequency of both the volume and slice acquisition are well below 50 Hz. Low-pass filtering of the data also strongly attenuates the harmonics of the volume and slice periods. This leads to a temporal jitter of the low frequency components of the artifact signal which in turn results in large residual artifacts after template subtraction. Therefore, low-pass pre-filtering alone is generally not recommended for artifact removal.

In the current application of FACET three different implementations of filters are offered. The selected filter and its specifications are applied to the data using PreFilter() (c.f. the complete listing of CleanEx1.m (Additional file 1) in the Additional files section for detail on available setting). These filters are: FIR (finite impulse response) filter, an ideal filter in the frequency domain, and a Gaussian filter, again in the frequency domain.

To realize a high attenuation in the narrow frequency band from 0 Hz to the cut-off frequency using FIR filters, a very high number of filter coefficients, here 1000, are used. This results in high demand of processing power. Additionally, the steep characteristic results in large overshoot of the transfer function, which contributes in turn to signal distortion effects.

While FIR filters work in the time domain, the frequency domain can be used to implement an ideal filter. This filter allows arbitrary modification of the amplitude of the frequency domain values of the signal. In the case of an ideal high-pass filter, all amplitude values below the cut-off frequency are set to 0.0. This approach gives the best attenuation, but results in severe ringing and oscillations after the inverse Fourier transform (iFFT) of the signal to time domain [22]. However, using an FFT and iFFT for these operations, leads to a dramatic reduction of the runtime of the filter compared to high-order FIR filters.

The maximum edge steepness of the frequency response without any overshoot is provided by Gaussian filters [23]. Although Matlab already provides a function to design a Gaussian FIR filter (gauss-fir) a Gaussian filter was reimplemented using a frequency domain filter because of its higher processing speed.

Due to the differences in the mean value (i.e., DC value) of the three sections of the EEG signal (before, during, and after fMRI acquisition), the filter is applied separately. Altogether, these filters can be either used as low-pass, as high-pass or as a combination of both. Additionally, methods to generate custom filters specified via a-priory chosen weights as well as the possibility to define individual cut-off frequencies per EEG channel are provided within FACET.

#### Setup of the correction algorithm

Most algorithms, in particular FMRI Artifact Slice Template Removal (FASTR) [17] and fMRI Artifact Reduction for Motion (FARM) [19], both used as a basis for this work, implement the individual correction steps in a fixed order. As already mentioned before, not every step is necessarily required, or additional steps or innovative combinations of present steps may need to be added. Therefore, the artifact correction algorithm was split into multiple blocks with a common interface for the input and output data. This allows these blocks to be flexibly combined and setup in an arbitrary order. Each block is implemented as a method of the FACET class. It operates on object member variables, which represent the EEG data (in successively improved quality) as well as the (reconstructed) artifact data. Using the usually large amount of data as a common (member) variable avoids the need to copy the whole dataset within each and every function, as would be required in a pure procedural design paradigm.

To configure which blocks are executed, a member variable RASequence is set to an array of strings (see Lst. 5). Each string specifies a corresponding block of the algorithm. These blocks are executed in exactly the given order. Additionally, user-defined functions can be included (note the entry @FACET.AvgArtWghtFARM), which are executed as well. Similar to the setup and analysis of the data, first an application example will be presented followed by a description of the different steps.

##### Correction algorithm example

**Figure 4:**
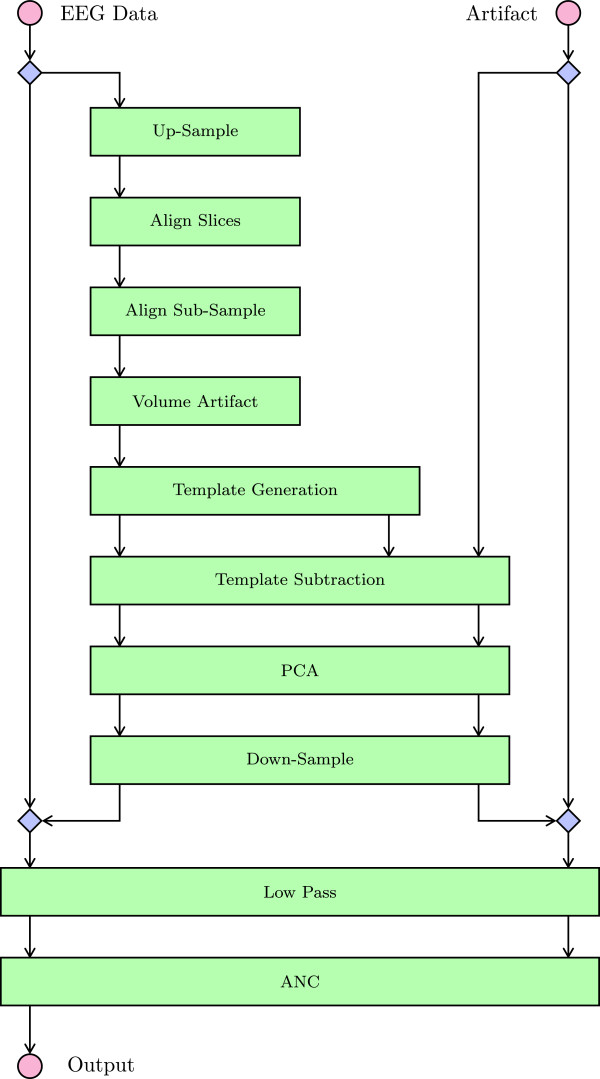
Artifact removal sequence as configured by Lst. 5.

Listing 5 shows an example as implemented by CleanEx1.m. In Figure 4 the corresponding data flow graph is given. The top blue diamond symbols denote the function ‘Cut’, which extracts the period of actual fMRI acquisition from the total EEG data for artifact estimation. This subarea of data is first up-sampled and further processed, including the template subtraction and PCA, before its sampling rate is reduced again. The bottom blue diamonds denote ‘Paste’, which merges the data back to the full EEG data. This is finally filtered with a low-pass filter and corrected using adaptive noise cancellation (ANC). Note that the (reconstructed) artifact signal (starting at the top right red circle) is initially zero, i.e. no artifact at all. The data is reconstructed step by step during the artifact removal.

##### Sub-sample alignment

**Figure 5:**
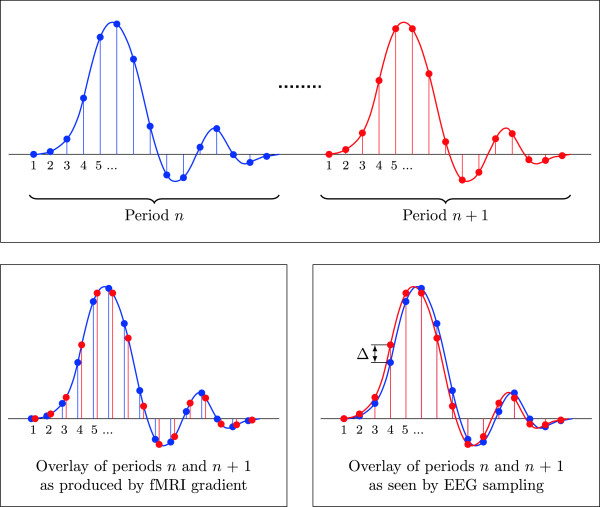
**Sampling by the EEG recording is not synchronized with the fMRI gradient signal.** Successive periods of the fMRI gradient signal are sampled by the EEG recording system with a varying temporal offset (top graph). Period *n* is drawn in blue and the following period *n*+1 in red. The bottom left graph shows these fMRI periods overlayed as sampled by the EEG recorder during period *n* (blue stems) and period *n*+1 (red stems). Overlaying the EEG samples at the quantized sampling times therefore leads to a vertical difference *Δ* for every period as denoted in the bottom right graph.

In most cases the fMRI scanner and the EEG recording use separate clocks and do not utilize synchronization. This means that, in general, every period of the MR gradient artifact is sampled by the EEG recording system with a varying temporal offset. This varying temporal offset is illustrated in Figure 5. Calculating an average of successive periods (i.e., epochs) leads to substantial error and thus residual artifact. To cope with this problem, two methods are provided. First, methods to increases the sampling rate by interpolating the data by a given factor (Upsample) and aligning the triggers within this finer temporal quantization (AlignSlices).

This reduces the residual amplitudes, but at steep slopes even a short uncertainty leads to large vertical differences (c.f., *Δ* in Figure 5 bottom right). While this method (up-sampling the data) helps to reduce the error, it also increases memory and processing demands.

Therefore, a second and recommended approach is to time-shift the data with a sub-sample resolution [19] utilizing the time shifting property of the Fourier transform (AlignSubSample). As a first step the EEG signal is converted to frequency domain using the FFT. Then a linear phase is added, depending on the desired sub-sample time-shift, before it is transformed back to the time domain using the inverse FFT. Since the necessary temporal shift is unknown, FACET implements an iterative optimization algorithm. This optimization minimizes the least square error of the current epoch to a (configurable) reference epoch using the bisection method.

##### Volume gap correction

The fMRI acquisition is performed volume by volume, therefore it shows a main period of the volume acquisition. For every volume a number of slices are acquired which pose a sub-period. Two cases for the relationship of slice and volume timing are possible: The time of a volume acquisition is an exact integer multiple of the time of the slice acquisition. In this case the acquisition of the last slice the fMRI scanner immediately continues with the acquisition of the first slice of the following volume. The second, more general, case exists if the acquisition of all slices lasts shorter than the repetition time of the volume acquisition. This results in a (short) delay between the acquisition of the last slice until the next volume starts with the acquisition of its first slice. Detection of these volume acquisition gaps is important since during this gap, an additional gradient artifact may be recorded and in addition, this artifact may extend to the following slice periods. This means that such slice periods have to be corrected with an additional method.

The algorithm implemented in FACET, called via RemoveVolumeArt, is similar to that used in FARM [19]: Assume an fMRI acquisition with *n*_*v*_ volumes of *n*_*s*_ slices each, i.e. a total of *n*_*v*_·*n*_*s*_=*n*_*e*_ slices. For every slice the time of its onset is given by *t*_*e*_ (with the epoch index *e*=1,…,*n*_*e*_). Due to the double periods, the epoch index can be expressed by the volume index *v*=1,…,*n*_*v*_and slice index *s*=1,…,*n*_*s*_ as *e*=(*v*−1)·*n*_*s*_+(*s*−1)+1^c^. This enables the trigger events to be written as *t*_*e*_=*t*_*v*,*s*_. With this notation, the volume gap exists between the last slice *s*=*n*_*s*_ of each volume *v* at $t_{v,n_{s}}$ and the first slice *s*=1 of the following volume *v*+1 at *t*_*v*+1,1_.

In a first step, the positions of the *n*_*v*_−1 volume gaps is determined by investigating the distances between the slice trigger events *t*_*e*_, utilizing the fact that

$$\begin{array}{cc} t_{v+1,1}-t_{v,n_{s}} & >t_{v,s+1}-t_{v,s}\;\;\forall \;v=1,\ldots ,n_{v}-1\;\text{and}\\ s & =1,\ldots ,n_{s}-1. \end{array}$$

As a second step, the volume artifact, which extends to the slice periods right next to the volume gap at $t_{v,n_{s}}$ and *t*_*v*+1,1_, is calculated. For this purpose the five slice epochs $t_{v,n_{s}-4}$ to $t_{v,n_{s}}$ before the volume gap and the five slice epochs *t*_*v*+1,1_ to *t*_*v*+1,5_ after the volume gap are averaged and subtracted from the adjacent slice periods at $t_{v,n_{s}}$ and *t*_*v*+1,1_, respectively.

These volume artifact templates are weighted with a logistic function

$$w_{x}=\frac{1}{1+e^{\alpha (x-x_{0})}}$$

to emphasize the artifact near the volume gap and de-emphasize it farther away. The parameters *α* and *x*_0_ are chosen to give a 50% weight at 80% of the slice interval, 10% at 69% of the interval and 1% at 57% of the interval. The weighted templates are then subtracted from the adjacent slice epochs.

In a final step, the gap is filled with a linear function from the end of the last slice $t_{v,n_{s}}$ to the beginning of the first slice of the next volume *t*_*v*+1,1_.

##### Artifact template matrix

The template generation step builds the core around every correction algorithm and usually calculates the average of surrounding artifact epochs. Depending on the exact algorithm, different epochs are selected for averaging. The selection of epochs and calculation of the average value is usually implemented using a loop which iterates over the epochs from the start to the end index value. A generalized approach, as implemented in FACET, uses a square matrix with the size equal to the number of epochs where the row and column indices correspond to the epochs [18].

Using a matrix to describe the averaging process poses the advantage that averaging can be performed with a single matrix multiplication. Let the raw EEG data of one channel be stored in the row vector *d*_*i*_ with *i*=1,…,*n*, where *n* is the total number of samples of the EEG recording. This vector is wrapped to the data matrix **D**=(*d*_*e*,*j*_), where each row *e*=1,…,*n*_*e*_ is one of the *n*_*e*_ epochs and *j*=1,…,*n*_*l*_ is the sample index within each epoch of length *n*_*l*_ (*n*_*e*_·*n*_*l*_=*n*).

The averaging matrix **A**=(*a*_*e*,*f*_) is a *n*_*e*_×*n*_*e*_ square matrix, where the columns *f* of every row *e* specify which epochs *f* are averaged (see above). The multiplication of the averaging matrix **A** with the data matrix **D** results in the noise (artifact) matrix **N**=(*n*_*e*,*j*_) with the same dimensions as **D**.

The final step is to wrap back the noise matrix **N** to a row vector like *d*_*i*_. Note that if the fMRI acquisition poses volume gaps (compare method RemoveVolumeArt), the estimated noise during these intervals is unknown. Two approaches are implemented by the artifact correction algorithm and setup with a configuration parameter. One method sets the noise values in the volume gaps to 0.0. The other method fills the gap with a linear function from the end of the last slice to the beginning of the first slice of the next volume, i.e., interpolated values are used. In contrast to the volume artifact correction detailed before, this interpolation is performed on the estimated noise data during template generation instead of the EEG input data during pre-processing.

##### Implemented artifact matrices

The beforehand described general averaging matrix provides a simple mean to calculate the templates in the template generation step (compare Figure 2). The matrix clearly shows which epochs are used for the templates. It is even possible to include weights, e.g. to enhance epochs near the current one while attenuating epochs further away. Therefore, any *linear combination* of the epochs is possible. Thus it represents a general and yet complete interface to specify an arbitrary template algorithm.

Numerous methods for the generation of this matrix to reflect specific averaging algorithms were implemented and added to the FACET package. These methods can be divided in adaptively calculated matrices and fixed defined calculation schemes. While the first group directly depends on the EEG data provided and therefore is defined within the RASequence, the second group does not depend on the data but solely on the number of slices and possible external data, like movement parameters.

Currently, two adaptively calculated averaging matrix methods are provided: FACET.AvgArtWghtFARM uses the best matching slice periods within a given window as used for the FARM algorithm [19] and FACET.AvgArtWghtAllen provides the functionality as implemented by AAS [16]. In FACET.AvgArtWghtAllen the template generation and subtraction is performed block-wise, i.e. templates are calculated from *n* consecutive epochs. According to [16]*n* is set to 25 epochs for periodic fMRI sequences with volume gaps and to 100 slices for sequences without delays between volumes. To further reduce the impact of atypical epoch signals, following the first five epochs the remaining epochs are added iteratively given their cross-correlation to the current template exceeded 0.975. Compared to this block-wise averaging, the template generation using FACET.AvgArtWghtFARM is improved by using a larger sliding window of 50 slices, but choosing a set of 12 artifacts with the highest correlation to the current artifact. Within FACET each of these parameters are predefined, but can be easily changed according to current needs. Besides these, a collection of data-independent methods is provided with the FACET package, implementing the behavior of FASTR [17], the realignment parameter informed algorithm [18] and others. Note that these are not integrated in the RASequence setting, since they can be executed before the algorithm itself (c.f. Lst. 6 on how to call the different approaches). For example, to reproduce FASTR’s behavior for volume and section triggers FACET.AvgArtWghtVolumeTrigger is used and for every second slice trigger FACET.AvgArtWghtSliceTrigger is used. For the calculation of a realignment parameter informed matrix similar to [18], e.g. FACET.AvgArtWghtMoosmann(E,’example01.txt’,0.8) can be used, assuming the file ’example01.txt’ contains the realignment parameters from SPM. Finally, a simple method to average the artifact template based on corresponding slices across the measured volumes can be assessed using with FACET.AvgArtWghtCorrespondingSlice(E,E.MRSlices) with the object MRSlice holding the acquisition sequence.

**Listing 6** Calculation of the averaging matrix for corresponding slices using an interleaved EPI-sequence. 

For all these adaptive and fixed methods the size of the averaging window can be defined using the AvgWindow property (see e.g. Lst. 1 or Lst. 6).

#### Post-processing of cleaned data

The afterwards described post-processing steps aim to further improve the EEG signal after removal of the gradient artifacts. These steps comprise of principal component analysis to remove the strongest components related to the fMRI artifact as introduced by [17], low-pass filtering the corrected signal to remove residual high-frequency components and final adaptive noise cancellation as used by [16]. Since these methods work only on the data related to the fMRI acquisition, they are therefore included into the RASequence (see Lst. 5 and Figure 4).

##### Principal component analysis

A temporal Principal Component Analysis (PCA) is used to find the functions which best describe the residual artifact signal, separately in each channel. The input data for the PCA is composed of only a random subset of all signal segments to avoid finding only artifacts which happen at regular multiples of the segments. From the principal components, the *C* strongest components (termed optimal basis set, OBS, by [17]) are used to build the estimated residual artifact. The number *C* is either configured by the user with the variable OBSNumPCs, or it is determined automatically during the algorithm performance by setting OBSNumPCs = ’auto’, based on the explained variance. The estimated residual artifact is finally scaled for best match with the signal and then subtracted.

##### Low-pass filter

As mentioned by [16], a low-pass filter as the only algorithm to reduce the artifacts does not suffice. On the other hand, it greatly improves the result as a post-processing step ([17]). Therefore the toolbox provides a low-pass filter with a configurable cut-off frequency using the filtfilt function implemented in Matlab.

As the filter is a post-processing step, it is only applied to the processed data, i.e., to the period with artifacts from fMRI acquisition, and not to the unimpaired EEG data before and after the fMRI acquisition. However, in certain situations one also wants to filter the data outside of the fMRI acquisition (i.e. the beginning and the end of the data). The behavior to use the full output of the algorithm, even outside of the fMRI acquisition area can be triggered by setting the variable DontTouchNonArtifact to “false” (c.f. the full listing of CleanedEx1.m).

##### Adaptive noise cancellation

As introduced by [16], Adaptive Noise Cancellation (ANC) [24] was also employed by [17]. The according source code of their FMRIB plugin (as well as the PCA code) is reused with only slight changes in FACET. The estimated artifact signal resulting from the previous steps is used as reference signal for the ANC. This implements an adaptive filter which is iteratively adjusted to remove the components which are correlated to the reference signal. The order of the ANC filter as well as its step size are determined automatically during the algorithm execution depending on properties of the EEG signal and the artifact.

### The evaluation framework

As an integral part of FACET, the evaluation framework allows the assessment of the quality of the results as soon as the EEG data with gradient artifacts is improved by the artifact correction algorithm. For this purpose, it provides an extensive set of performance indicators as utilized by different authors.

The goal was to perform multiple evaluation algorithms on the output data from the artifact removal algorithms. These evaluation results can be saved and then presented to the user in several formats. One format is to print the numbers with some descriptive text to the screen. Additionally, LATE X source code can be used to produce tables and diagrams for high-quality publications.

**Figure 6:**
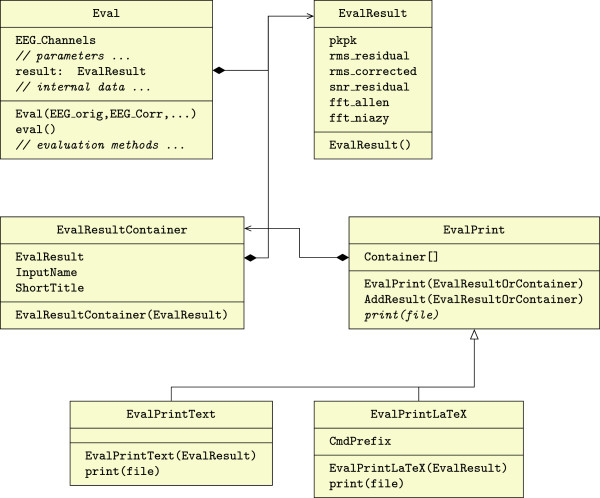
UML Class Diagram of the evaluation framework.

For the evaluation framework, the same modern design paradigm of object oriented programming as for the correction algorithm is used, i.e. the software design provides a separation of (1) the execution of performance indicator algorithms, from (2) the storage of their results, and (3) the presentation of these results. This is achieved by the object-oriented design as shown in Figure 6. The functionality is divided and realized by the classes. Eval is the main class which holds all evaluation algorithms. It is instantiated with the original and the corrected EEG data (EEGLAB data structure). The evaluation is performed by the method eval(). The results are stored in the member variable result, which is an instance of the EvalResult class. EvalResult is a storage object for the evaluation results. EvalResultContainer wraps the EvalResult object and adds meta information (the variable name and a short title), later used by the EvalPrint descendants. EvalPrint is an abstract base class, which is used to print the evaluation results in an arbitrary format. Actual printing routines are implemented in descendant classes (see below). EvalPrintText is a descendant class of EvalPrint which implements the print() method to display a human-readable table. EvalPrintLaTeX also descends from EvalPrint and implements the creation of output to be used in LATE X documents. It generates code using the Ti*k*Z and PGFPlots packages with multiple commands, which are used by the user inside of table and figure environments.

#### Performance indicators

To assess the quality of results after the application of a correction algorithm, multiple criteria found in previous publications were employed. These indicators can be grouped according to their underlying physical or numerical property into amplitude criteria like median signal amplitude, RMS and SNR criteria and criteria within the frequency domain.

##### Median imaging artifact

This indicator calculates the median signal amplitude range across all channels The amplitude of the artifact is used by [16] as an indicator of the quality of the AAS algorithm. To calculate this measure ten intervals with a length of 10% to 20% more than the slice period time are taken from the EEG data of every channel. These intervals are equally spaced to cover the whole duration of the fMRI acquisition. The range (maximum value minus minimum value) of each interval is calculated and averaged across the ten samples. This results in a mean imaging artifact range (also called peak-to-peak value) of every channel within the recording. Finally the minimum, maximum and median of these ranges are calculated, where the latter is called “Median Imaging Artifact”.

Although [16] solely use the median imaging artifact to show the reduction of the residual artifact, we also calculate the median range of the EEG data without fMRI acquisition for comparison. Therefore the very same algorithm is performed on the (merged) EEG data before and after fMRI acquisition.

##### RMS ratios

The Root-Mean-Square value (RMS) is the quadratic mean of a signal and is defined for a continuous time signal *x*(*t*) and discrete time signal *x*_*i*_ by

$$x_{\text{RMS}}=\sqrt{\frac{1}{T}\int_{0}^{T}x^{2}(t)\text{dt}}=\sqrt{\frac{1}{n}\overset{n}{\underset{i=1}{\sum }}x_{i}^{2}},$$

respectively. Within FACET two RMS measures are implemented, the “RMS Corrected to Unimpaired.” and the “RMS Uncorrected to Corrected”. The “Corrected to Unimpaired” performance indicator used by [18] is the ratio of the RMS of the (DC-free) corrected EEG signal to the RMS of the signal without fMRI acquisition. This performance indicator represents the fraction of the corrected signal to the pure signal. If the ratio is greater than 1, residual artifacts are still present while values below 1 show that the correction algorithm removed parts of the wanted signal. “RMS Uncorrected to Corrected” proposed by [19] use the ratio of the RMS of the uncorrected EEG signal to the RMS of the corrected signal. The bigger this ratio the better, i.e., the more artifact was removed. However, non of these measures show whether the algorithm has removed too much of the signal. The evaluation tool calculates both ratios individually for every channel and returns descriptive statistics on them.

##### SNR of corrected

The Signal to Noise Ratio (SNR) commonly relates the undistorted signal power *S* to the noise power *N*. In many cases these values are not directly available for practical calculations. Examples are the EEG signal from an fMRI acquisition or the result from the artifact correction algorithm.

In these cases only the power of the distorted signal *D* is known. Assuming additive noise, the distorted signal can be written as sum of the undistorted signal and the noise signal *x*_*D*_(*t*)=*x*_*S*_(*t*)+*x*_*N*_(*t*). Utilizing the statistical independence, the power of the undistorted signal and the noise signal are also additive: *D*=*S*+*N*.

Most EEG datasets with fMRI acquisition also contain periods without fMRI acquisition. These can be used as a sample of the undistorted signal to calculate *S*. With this information, the noise power is given by *N*=*D*−*S* and the SNR is

$$\text{SNR}=\frac{S}{D-S}.$$

This equation is applied for every channel of the corrected EEG dataset with *D* as the corrected signal and *S* the signal outside of the fMRI acquisition periods.

Due to the subtraction term *D*−*S* the result can be negative. This happens when the correction algorithm has removed more signal power than the artifact signal power. Physically a negative noise power *N*=*D*−*S* does not make sense, therefore only positive SNR values are considered in the descriptive statistics.

##### Median residual activity

This performance indicator is a measure for the deviance of the corrected signal in different EEG frequency bands [16]. To compare the activity in four EEG frequency bands (0.8–4 Hz: Delta waves, 4–8 Hz: Theta waves, 8–12 Hz: Alpha waves, and 12–24 Hz: part of the Beta wave spectrum), ten equally spaced periods of 3 sec. (which are 6.000 samples at a sampling rate of 2 kSps) are averaged and then transformed to frequency domain with an FFT for every channel. Then the mean activity in every frequency band of the corrected signal (*A*_corr_) is compared to the mean activity during no fMRI acquisition (*A*_NI_) by calculating the absolute value of the relative difference in percent

$$\text{Percentage Difference}=100\%\times \left|\frac{A_{\text{corr}}-A_{\text{NI}}}{A_{\text{NI}}}\right|.$$

To summarize the percentage difference values, the median (due to non-normality) for all channels is determined and given separately for each frequency band.

##### Power density at slice frequency

This final frequency specific performance indicator lists the power density reduction achieved by the artifact correction at the slice frequency and its harmonics. For the given original and the corrected datasets an FFT is performed for every channel. The magnitudes at the frequency bins of the volume and the slice frequencies and four harmonics are divided (corrected /original) and squared. These values are converted to decibel (dB) and the mean over all channels is calculated.

The automatic generation of LATE X source code by EvalPrintLaTeX for clarity only includes a subset of the evaluation results. For the “Median Imaging Artifact” the median amplitude range of the corrected data is shown. The “RMS Corrected to Unimpaired”, the “RMS Uncorrected to Corrected” as well as the “SNR of Corrected” are represented by the respective mean values across all channels. The mean SNR value is supplemented by the number of included values with a positive noise power. For the “Power Density at Slice Frequency” the averaged value in dB of the slice frequency and its harmonics across all channels are given.

#### Usage examples

For a quick evaluation of the results, the evaluation framework is usable with a one line Matlab command. The example in Lst. 7 compares the corrected dataset cleaned1 to the raw dataset EEG_FMRIB, but limits the comparison to channels 1 to 30 (because channel 31 contains ECG data, see below). Additionally, the number of slices per volume is given as 21. The command-line creates an Eval object and executes its eval() method. Then it creates an EvalPrintText object and print()s the evaluation result to the screen. The evaluation result is stored to the variable eval1.

**Listing 7** Exemplary one-line Matlab command to evaluate the result of the correction algorithm and to print the result.

For a more complex case, it is advisable to store the Eval, EvalResult and EvalPrint* objects to variables and access their member variables for fine tuning.

An example is given in Lst. 8. In the first part, datasets are evaluated, each corrected once using Averaged Artifact Subtraction (AAS) and once using FMRI Artifact Slice Template Removal (FASTR). In the second part, an EvalPrintLaTeX object is created. The evaluation results are added and short labels are provided. A prefix for the generated LATE X commands is given, before the result is written to a file. This can be used in a LATE X document, which flexibly places the generated tables and diagrams. This feature was extensively used to prepare all evaluation figures found within the next section.

**Listing 8** Example of the usage of the evaluation framework to generate the LATE X tables and diagrams for the AAS and FASTR algorithm found in this paper.

## Results

Having described the possibilities and implemented methods of FACET the following section will demonstrate the functionality of FACET using the dataset supplied with the FMRIB plugin by Niazy et al. [17]^d^. This dataset was recorded using a SystemPLUS EEG system and an SD32 MRI amplifier by Micromed s.r.l., TV, Italy and consists of EEG data acquired from 32 channels: 30 EEG-channels complemented by two bipolar channels for electromyography (EMG) and electrocardiography (ECG). Concurrent with the EEG data 40 EPI volumes (TR=3000 ms and 21 slices per volume) were acquired using a 3 T Varian Inova scanner. The subject’s task was to open and close his eyes every 10 seconds. Listing 2 shows further details on the EEG dataset as found with the AnalyzeData() method.

We will begin with a short characterization of the two algorithms used by Allen [16] and Niazy [17]. Then we will demonstrate the functionality of the CORRECTION framework to clean the given dataset using these two algorithms. Finally, the obtained results will be evaluated using the EVALUATION framework of FACET. All scripts used to demonstrate the functionality of FACET are provided with the FACET package as well as in the appendix (Additional file 2).

### Description of the correction algorithms

The averaged artifact subtraction (AAS [16]) algorithm utilizes the periodic shape of the artifact signal to calculate an artifact template. To circumvent high residual artifacts due to signal drifts or subject movements the template generation and subtraction is performed block-wise. To further reduce the impact of atypical epoch signals, following the first five epochs, remaining epochs are added iteratively provided that their cross-correlation to the current template exceeds 0.975. Residual artifacts are then eliminated using adaptive noise cancellation (ANC). The problem of missing synchronization of EEG and MR is tackled here by up-sampling using a sinc-interpolation before template calculation.

The FMRI artifact slice template removal (FASTR) algorithm was introduced by [17,25]. Similar to AAS, it uses triggers from the MR scanner which indicate the slice timing. However, in this case, the triggers have the same temporal resolution as the EEG sampling, as opposed to those used by AAS [16], which have a higher temporal resolution. To tackle the problem of unsynchronized acquisition, the EEG data is up-sampled, similar to AAS, then MR-trigger indices are adjusted within the refined time resolution. The artifact template is calculated as a local moving average of a configurable number of slice segments. To remove residual artifacts a set of basis functions using the principal component analysis (PCA) method is calculated and components describing most of the variance (called optimal basis set, OBS, here), are scaled and added to the artifact template signal from the previous stage. Similar to Allen [16] adaptive noise canceling (ANC) is used with the reference being the estimated error signal.

### Setup of the evaluation

The FMRIB dataset was used as input data for three different algorithms. Their results were then evaluated using the EVALUATION framework of FACET. Both aforementioned correction algorithms, averaged artifact subtraction (AAS) and FMRI artifact slice template removal (FASTR) were implemented with the FACET CORRECTION framework using the same parameters as proposed in [16] and [17]. The complete listing for AAS (CleanAAS.m) and FASTR (CleanFASTR.m) together with the evaluation script (EvaluateFacet.m) can be found in the additional files section (Additional file 2). FASTR as implemented with FACET is termed FASTR-F to differentiate it from the the original FASTR implementation (termed FASTR-N) which was also used for comparison.

For the AAS algorithm, the setup parameters are: 

● Length of averaging window: 100 consecutive slices

● Interpolation factor: 10

● Cross-correlation to the current template: 0.975

● Low-pass filter cutoff: 70 Hz

The FASTR algorithm was implemented using: 

● High-pass filter cutoff: 1 Hz

● Length of averaging window: 30 slices, 15 slices before and after the current one. Only every second slice is used so that the EEG signal is uncorrelated between slices.

● Interpolation factor: 10

● Low-pass filter cutoff: 70 Hz

Divergent to the original publications, we did not use a sinc-interpolation before template calculation but the interp function as provided by Matlab for the AAS algorithm. Furthermore, a 70 Hz low-pass filter was applied after artifact removal to better allow for comparability of the different algorithms. For FASTR-F the sequence of the artifact removal was slightly changed. While the calculation of the optimal basis set is similar to the original FASTR-plugin, the resulting OBS was not added to the averaging matrix but subtracted in a separate step. Additionally, we used a Gaussian filter in the frequency domain as implemented in FACET. For both algorithms, c.f. [17], the noise signal was used as reference for the adaptive noise cancellation step and not the low-passed version of a binary vector reflecting the slice timing as done by [16].

### Results of the evaluation

**Figure 7:**
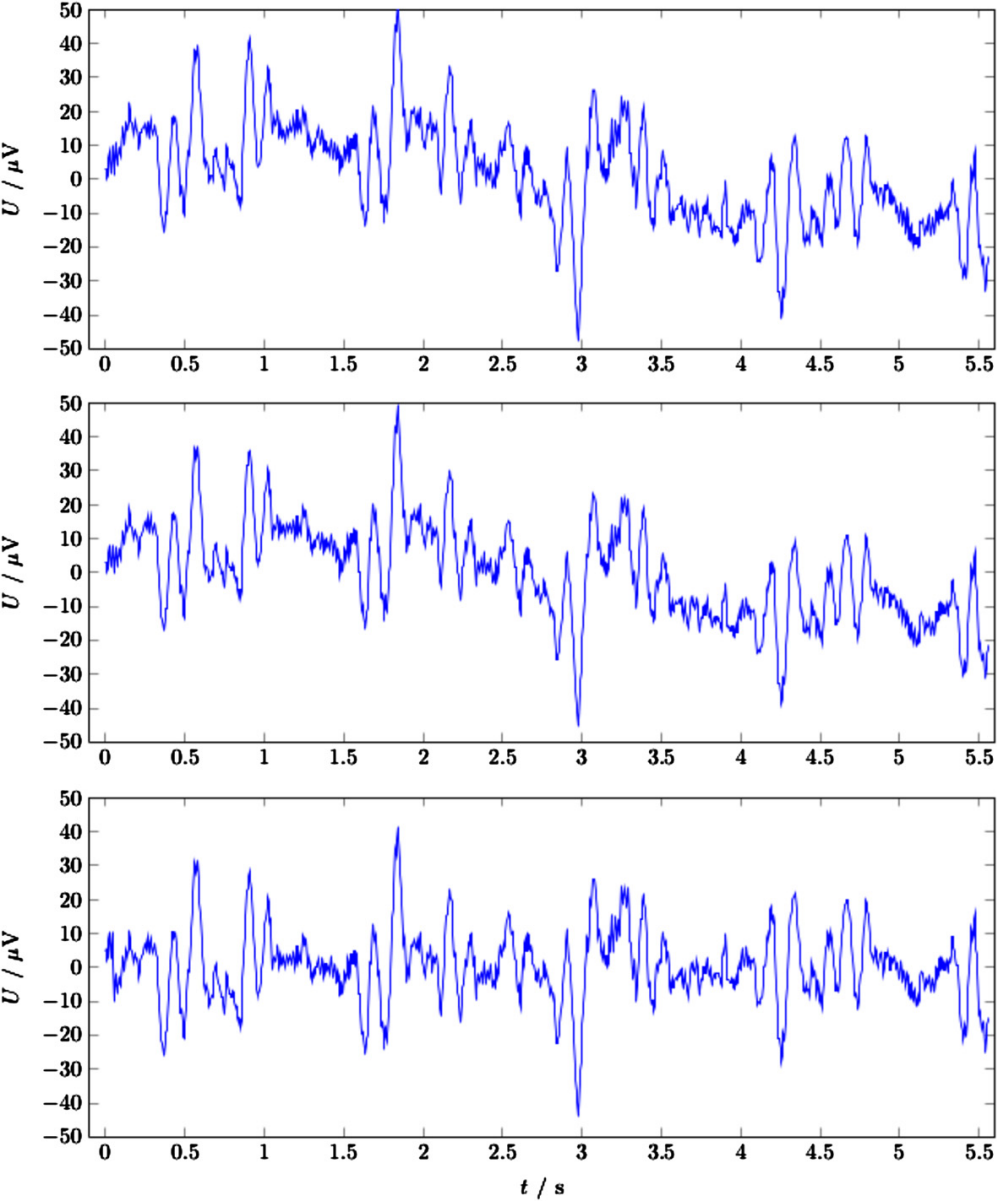
**Exemplary section of the FMRIB dataset showing the time course at channel Fp1 for approximately 2 volumns (42 slices) after artifact removal with the AAS (top), FASTR (middle, FASTR-N) and FASTR as implemented in FACET (bottom, FASTR-F) algorithm.** The signal does not show any slice or volume artifacts. The residual variation is due to the BCG artifact which is currently not considered in FACET.

Figure 7 shows a section of the FMRIB dataset corresponding to 42 slices or 2 volumes after correction using the three different algorithms. This figure shows that the gradient artifact was successfully corrected and none of the three signals show any slice or volume artifacts. The residual variation is due to the ballistocardiographic artifact (BCG) which is currently not considered in FACET.

**Figure 8:**
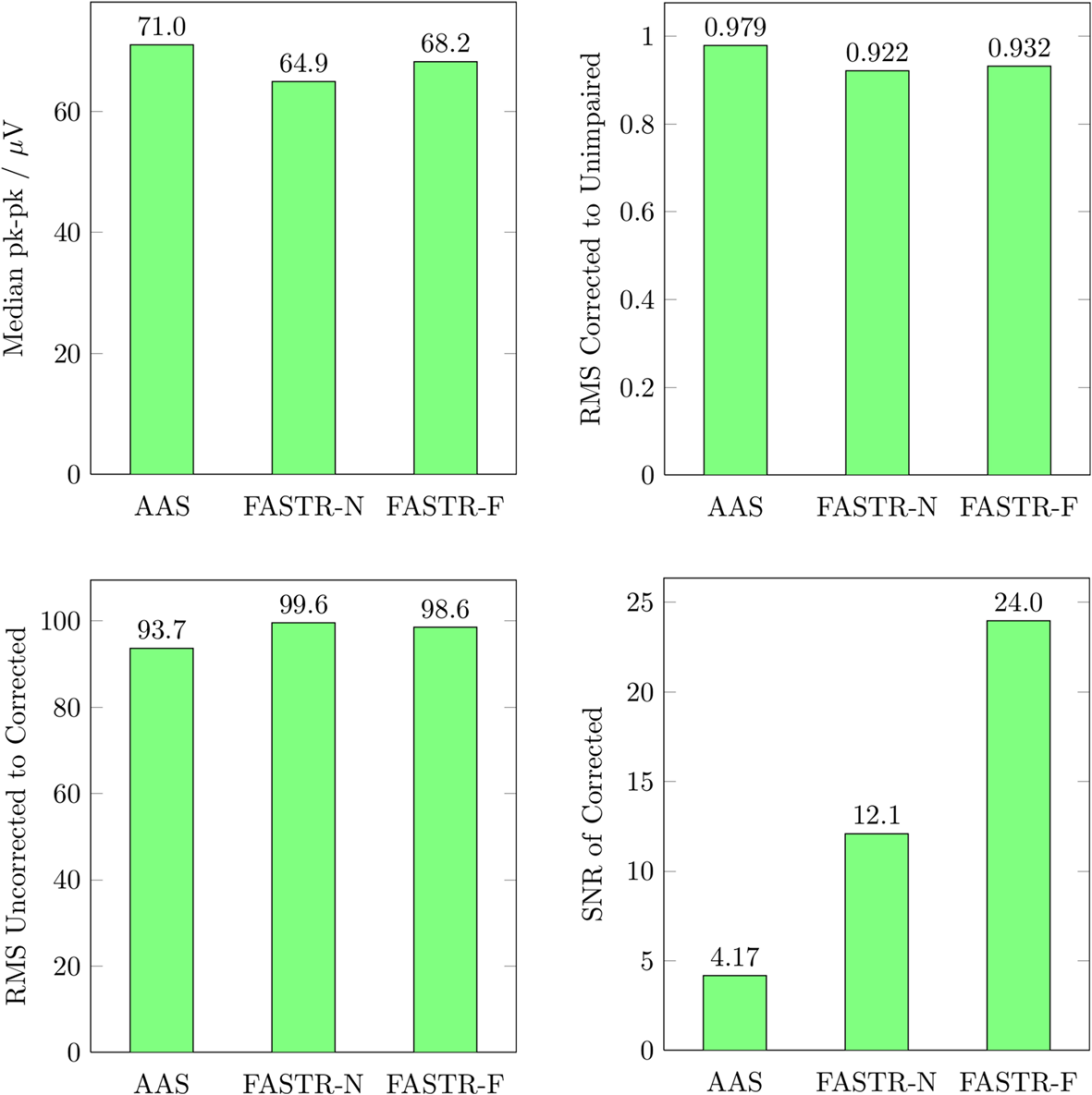
**Comparison of the results of AAS, FASTR-N and FASTR-F using time-based measures.** Top left: the median imaging artifact (lower is better), top right: RMS of the corrected to the unimpaired signal (nearer to 1.0 is better), bottom left: RMS of the uncorrected to the corrected signal (larger is better) and bottom right: SNR of the corrected signal (larger is better).

**Figure 9:**
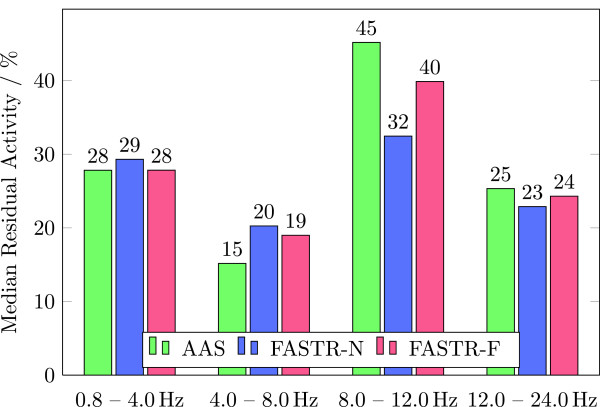
Comparison of the results of AAS, FASTR-N and FASTR-F by the median residual activity within different frequency bands (lower is better).

**Figure 10:**
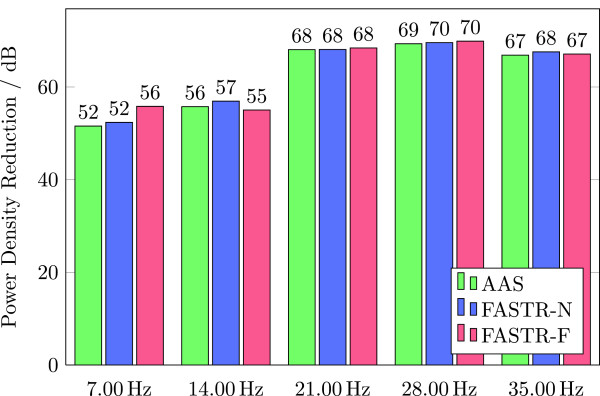
Comparison of the results of AAS, FASTR-N and FASTR-F by the power density reduction at slice frequency harmonics (larger is better).

While the signals do not evince obvious differences between the used correction algorithms, individual evaluation summarized in Table 1 and presented in Figures 8, 9, and 10 clearly favor the FASTR over the AAS algorithm. Most performance indicators in the time-domain (median imaging artifact, RMS, SNR) show an advantage of FASTR over AAS; e.g. the median image artifact shows a decrease from 71 *μ*V for AAS to 64.9 *μ*V and 68.2 *μ*V for FASTR-N and FASTR-F, the ratio of the RMS of the uncorrected EEG signal to the RMS of the corrected signal shows an increase from 93.7 (AAS) to 99.6 (FASTR-N) and 98.6 for FASTR-F as implemented in FACET.

**Table 1:** Evaluation results comparing AAS, FASTR implemented in the FMRIB plugin (FASTR-N) and FASTR implemented in FACET (FASTR-F)

	Field MR Centre of Excellence, Medical University of Vienna, Lazarettgasse 14, A-1090 Vienna, Austria **AAS**	**FASTR-N**	**FASTR-F**
Median imaging artifact (*μ*V)	71.0	64.9	68.2
RMS corrected to unimpaired	0.979	0.922	0.932
RMS uncorrected to corrected	93.7	99.6	98.6
SNR of corrected	4.17 (11)	12.1 (9)	24.0 (10)
Median residual activity			
0.8–4.0 Hz	28%	29%	28%
4.0–8.0 Hz	15%	20%	19%
8.0–12.0 Hz	45%	32%	40%
12.0–24.0 Hz	25%	23%	24%
Power density at slice frequency			
1: 7.00 Hz	-52 dB	-52 dB	-56 dB
2: 14.00 Hz	-56 dB	-57 dB	-55 dB
3: 21.00 Hz	-68 dB	-68 dB	-68 dB
4: 28.00 Hz	-69 dB	-70 dB	-70 dB
5: 35.00 Hz	-67 dB	-68 dB	-67 dB

On the other hand, the frequency domain performance indicators show a partial deterioration of the median residual activity in the alpha frequency bands (8–12 Hz) for the AAS and the FASTR-F algorithms. While this band does not contain slice frequency harmonics (7 Hz, 14 Hz, 21 Hz, 28 Hz,...), these are present in the 4–8 Hz and 12–24 Hz bands, which both show almost equal performance parameters between the three correction algorithms. The power density reduction at slice frequency harmonics again shows equal and comparable results across all three algorithms.

**Figure 11:**
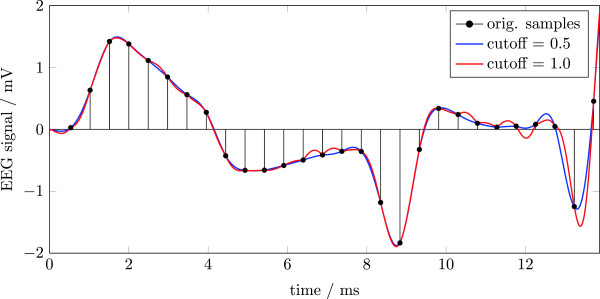
**Setting the cut-off frequency parameter of **Matlab**’s **interp **function to 1.0 results in deviations of the up-sampled data (red curve) from the smooth signal (blue curve) between the original samples (black stems).**

Direct comparison of the two implementations of the FASTR algorithms, once using the FMRIB plugin (FASTR-N) and once using FACET (FASTR-F), shows equal and comparable evaluation results with respect to the gradient artifacts. Only the Signal to Noise Ratio (SNR) relating the undistorted signal power to the noise power is doubled, c.f. Figure 8, in the FACET implementation. The reason for this discrepancy might be manifold, but is most likely due the slightly different implementation of the sequence of the artifact removal. Within the original FASTR implementation, results from the OBS are added to the averaging matrix. This might result in some unwanted cancellations or elevation of the final noise signal, possibly leading to the increased SNR within FASTR-F. Another possibility for the observed differences might be the different settings for relative cut-off frequency parameters for the interpolation filter which is set to 1.0 in the FMRIB plugin and to 0.5 in FACET (see also Figure 11) and the Gaussian high-pass applied to the whole dataset and not only for artifact filtering. This last change to the original algorithms is also the cause for the missing overall slow negative drift of the signal in Figure 7, bottom window.

Finally, although not a quality criterion for artifact correction but a quality criterion for the implementation within FACET, we evaluated the processing time needed to run the different algorithms. While FASTR using the FMRIB plugin executed for 2:08 minutes, implementing this algorithm in FACET almost halved the processing time to 1:06 minutes. The AAS algorithm, having the least processing steps, took 50.9 seconds to complete.

## Discussion

Multi-modal imaging is an aspiring methodology in neuroscience. It enables research as well as clinical applications to observe brain activity with distinct approaches and allows to correlate results of both. The application of concurrent EEG, EMG and fMRI poses problems due to mutual interference. While the artifacts in fMRI data due to EEG electrodes and equipment is negligible, the fMRI gradient artifacts in the EEG data caused by electromagnetic induction exceed the original EEG signal by several orders of magnitude.

Within every channel, the artifact signal shape is a periodic function for the acquisition of every fMRI slice and/or volume. Many algorithms utilize this fact to calculate an artifact template (e.g. by averaging all slice artifacts) and subtract this from the original data (averaged artifact subtraction: AAS [16]). This template subtraction paradigm still leaves residual artifact signals which are larger than the biological signals. These residual artifacts originate from missing synchronisation between the fMRI acquisition and the EEG sampling, slow drifts of the artifact shape, or sudden movements of the subjects among others. Consequently, the calculation of the template is optimised and several pre-processing and post-processing steps are added by many authors.

Several mostly multi-step correction algorithms were published tackling either one or several of the beforehand mentioned issues to improve the correction. However, these algorithms often apply the individual steps in fixed order leaving no space for the flexibility to either leave out or add new steps. Furthermore, not all correction algorithms are freely available to the scientific community but are either proprietary or simply not available.

In this paper we presented FACET – a “Flexible Artifact Correction and Evaluation Toolbox” for the correction of gradient artifacts in concurrently recorded EEG/fMRI data. FACET is implemented in Matlab and relies on the EEGLAB data structure [21]. The whole toolbox consists of an ANALYSIS, a CORRECTION and an EVALUATION framework. The ANALYSIS framework provides information on the EEG data at hand. Within the CORRECTION framework a selection of various algorithms for correcting imaging induced artifacts are provided. Additionally, various pre- and post-processing steps are implemented like volume onset detection, sub-sample alignment or OBS and ANC. All steps are implemented in a modular fashion to allow flexible combinations of different approaches. The EVALUATION framework of FACET allows the assessment of the quality of the chosen correction approach and a comparison between different settings. In total, the “FACET” toolbox provides facilities for all three: data analysis, artifact correction as well as evaluation and documentation of the results.

This toolbox was used to correct and evaluate a publicly available dataset using two popular and widely used gradient correction algorithms: the Averaged Artifact Subtraction (AAS [16]) and the FMRI Artifact Slice Template Removal (FASTR [17]) algorithm. In addition the FMRIB dataset was corrected using the FASTR implementation provided with the EEGLAB plugin FMRIB and then evaluated using FACET. As expected FASTR outperformed the AAS algorithm across all performance measures FASTR, irrespective which implementation was used. A direct comparison of the two FASTR implementations showed an improvement in the processing time by a factor of almost two while all evaluation measures related to the gradient artifact showed equal performance.

In summary, the presented artifact correction and evaluation toolbox provides an extensible and flexible tool for the scientific community. It employs well known data structures using EEGLAB and a simple, yet powerful, method to setup the algorithm in almost any sequence which also ease the integration of improvements. FACET is provided at https://github.com/hansiglaser/facet under the terms of the GNU General Public License (GPL) to provide powerful collaboration, review, and code management of possible future extensions.

## Future work

Extensibility of the toolbox was one of the design goals of FACET and is achieved with the object oriented design paradigm. Therefore, several improvements of FACET are planned. Among these is the correction of ballistocardiographic (BCG) artifacts which is currently missing. Further improvements concern enhanced usability of the toolbox, in particular we plan to extent the CheckData() method to check all sensible settings for validity and interdependencies.

Since the EEG signals and fMRI gradient artifacts as well as the BCG are mutually statistically independent, an independent component analysis (ICA) can be added to further improve the gradient template estimation before subtraction. A very promising approach in this direction was recently shown in [26].

Other interesting enhancements are the usage of the Wavelet transform for the artifact correction [27]. Contrary to Wavelet shrinkage, which is used to remove the small signal components, here the large signal components should be removed. This could be for example achieved by an inverted thresholding function.

Finally, during the execution of the algorithm, an identical sequence is performed on the data of every EEG channel. This allows for easy parallelization using multi-core CPUs or computing clusters. Newer versions of Matlab also allow the transfer of large computational tasks to graphics processing units (GPUs), which are highly optimized engines for parallel processing.

## Conclusion

In summary, the “FACET” toolbox provides facilities for all three modalities: data analysis, artifact correction as well as evaluation and documentation of the results. Due to its flexible and modular structure any combination of the implemented processing steps can be selected individually in any sequence to create new processing pipelines. The results then can be evaluated and compared to existing approaches within the same toolbox. Thus, FACET not only provides a valuable tool for the removal of imaging artefacts from concurrently recorded EEG and EMG data but also offers an easily extendable framework for development and evaluation of new approaches.

## Availability and requirements

● **Project name:** FACET (Flexible Artifact Correction and Evaluation Toolbox)

● **Availability:** Public GitHub: https://github.com/hansiglaser/facet

● **Operating system:** Platform independent (FACET was tested on Linux and Windows platforms)

● **Programming language:** MATLAB (FACET was tested on R2010b and later releases)

● **Requirements:** FACET relies on the EEGLAB data structure [21] available at http://sccn.ucsd.edu/eeglab/. To run the example scripts, one also needs to install the FASTR algorithm [17] available as an EEGLAB plugin available at http://www.fmrib.ox.ac.uk/eeglab/fmribplugin.

● **Licence:** This program is free software; you can redistribute it and/or modify it under the terms of the GNU General Public License as published by the Free Software Foundation; either version 2 of the License, or (at your option) any later version the terms of the GNU General Public License (GPL) to provide powerful collaboration, review, and code management of possible future extensions.

## Endnotes

^a^EEGLAB available at http://sccn.ucsd.edu/eeglab/

^b^ FASTR plugin available at http://www.fmrib.ox.ac.uk/eeglab/fmribplugin

^c^ −1 for *v* and *s* as well as +1 for the whole formula are necessary because the indices start at 1

^d^ This dataset is freely available at http://www.fmrib.ox.ac.uk/eeglab/fmribplugin/

## Competing interests

The authors declare that they have no competing interests.

## Authors’ contributions

JG programmed the toolbox and wrote the section on the implementation of the toolbox. RB and HB co-wrote the manuscript and evaluated the toolbox. FF co-designed the toolbox and implemented the usage examples and wrote large parts of the manuscript. All authors read and approved the final manuscript.

## Supplementary Material

Additional file 1 **Complete listing of cleanEx1.m.** This file contains a complete listing of the example script CleanEx1.m including a detailed documentation of the various settings. Click here for file

Additional file 2 **Evaluation scripts as compressed zip-file.** This Zip-file contains the complete listings of CleanAAS.m, CleanFASTR.m implementing different correction approaches and EvaluateFACET.m used to generate the evaluation results and figures presented within this manuscript. Click here for file

## References

[B1] Biessmann F Plis S Meinecke FC Eichele T Müller KR Analysis of multimodal neuroimaging data IEEE Rev Biomed Eng 2011 4 26 58 22273790 10.1109/RBME.2011.2170675

[B2] Ives J Warach S Schmitt F Edelman R Schomer D Monitoring the patient’s EEG during echo planar MRI Electroencephalogr Clin Neurophysiol 1993 87 6 417 420 10.1016/0013-4694(93)90156-P 7508375

[B3] Sadaghiani S Scheeringa R Lehongre K Morillon B Giraud AL Kleinschmidt A Intrinsic connectivity networks, alpha oscillations, and tonic alertness: a simultaneous electroencephalography/functional magnetic resonance imaging study J Neurosci Official J Soc Neurosci 2010 30 30 10243 10250 10.1523/JNEUROSCI.1004-10.2010 PMC6633365 20668207

[B4] Scheeringa R Petersson KM Kleinschmidt A Jensen O Bastiaansen MCM EEG alpha power modulation of FMRI resting-state connectivity Brain Connectivity 2012 2 5 254 264 10.1089/brain.2012.0088 22938826 PMC3621304

[B5] Kaufmann C Wehrle R Wetter TC Holsboer F Auer DP Pollmächer T Czisch M Brain activation and hypothalamic functional connectivity during human non-rapid eye movement sleep: an EEG/fMRI study Brain J Neurol 2006 129 Pt 3 655 667 10.1093/brain/awh686 16339798

[B6] Dang-Vu TT Schabus M Desseilles M Albouy G Boly M Darsaud A Gais S Rauchs G Sterpenich V Vandewalle G Carrier J Moonen G Balteau E Degueldre C Luxen A Phillips C Maquet P Spontaneous neural activity during human slow wave sleep Proc Natl Acad Sci USA 2008 105 39 15160 15165 10.1073/pnas.0801819105 18815373 PMC2567508

[B7] Vulliemoz S Lemieux L Daunizeau J Michel CM Duncan JS The combination of EEG source imaging and EEG-correlated functional MRI to map epileptic networks Epilepsia 2010 51 4 491 505 10.1111/j.1528-1167.2009.02342.x 19817805

[B8] Chaudhary UJ Carmichael DW Rodionov R Thornton RC Bartlett P Vulliemoz S Micallef C McEvoy AW Diehl B Walker MC Duncan JS Lemieux L Mapping preictal and ictal haemodynamic networks using video-electroencephalography and functional imaging Brain 2012 135 12 3645 3663 10.1093/brain/aws302 23250884

[B9] Menon V Crottaz-Herbette S Combined EEG and fMRI studies of human brain function Int Rev Neurobiol 2005 66 291 321 16387208 10.1016/S0074-7742(05)66010-2

[B10] Yan WX Mullinger KJ Geirsdottir GB Bowtell R Physical modeling of pulse artefact sources in simultaneous EEG/fMRI Human Brain Mapping 2010 31 4 604 620 19823981 10.1002/hbm.20891 PMC6870628

[B11] Yan C Liu D He Y Zou Q Zhu C Zuo X Long X Zang Y Spontaneous brain activity in the default mode network is sensitive to different resting-state conditions with limited cognitive load PloS one 2009 4 5 e5743 10.1371/journal.pone.0005743 19492040 PMC2683943

[B12] Mullinger KJ Yan WX Bowtell R Reducing the gradient artefact in simultaneous EEG-fMRI by adjusting the subject’s axial position NeuroImage 2010 10.1016/j.neuroimage.2010.09.079 PMC3095086 20932913

[B13] Laufs H Daunizeau J Carmichael DW Kleinschmidt A Recent advances in recording electrophysiological data simultaneously with magnetic resonance imaging NeuroImage 2008 40 515 528 10.1016/j.neuroimage.2007.11.039 18201910

[B14] Ritter P Becker R Graefe C Villringer A Evaluating gradient artifact correction of EEG data acquired simultaneously with fMRI Magnet Reson Imaging 2007 25 6 923 932 10.1016/j.mri.2007.03.005 17462844

[B15] Ullsperger M Debener S Simultaneous EEG and fMRI: Recording, Analysis, and Application 2010 USA: Oxford University Press

[B16] Allen PJ Josephs O Turner R A method for removing imaging artifact from continuous EEG recorded during functional MRI NeuroImage 2000 12 2 230 239 10.1006/nimg.2000.0599 10913328

[B17] Niazy RK Beckmann CF Iannetti GD Brady JM Smith SM Removal of FMRI environment artifacts from EEG data using optimal basis sets NeuroImage 2005 28 3 720 737 10.1016/j.neuroimage.2005.06.067 16150610

[B18] Moosmann M Schönfelder V Specht K Realignment parameter-informed artefact correction for simultaneous EEG-fMRI recordings NeuroImage 2009 45 4 1144 1150 10.1016/j.neuroimage.2009.01.024 19349230

[B19] Van Der Meer JN Tijssen MAJ Bour LJ Rootselaar AFV Nederveen AJ van der Meer JN van Rootselaar AFF Robust EMG-fMRI artifact reduction for motion (FARM) Clin Neurophysiol Official J Int Fed Clin Neurophysiol 2010 121 5 766 776 10.1016/j.clinph.2009.12.035 20117046

[B20] Mandelkow H Brandeis D Boesiger P Good practices in EEG-MRI: The utility of retrospective synchronization and PCA for the removal of MRI gradient artefacts NeuroImage 2010 49 3 2287 2303 10.1016/j.neuroimage.2009.10.050 19892021

[B21] Delorme A Makeig S EEGLAB: an open source toolbox for analysis of single-trial EEG dynamics including independent component analysis J Neurosci Methods 2004 134 9 21 10.1016/j.jneumeth.2003.10.009 15102499

[B22] Oppenheim AV Willsky AS Nawab SH Signals & Systems, 2 edition 1997 Upper Saddle River: Prentice-Hall International Inc.

[B23] Rappaport TS Wireless Communications: Principles and Practice 2001 Upper Saddle River, London: Prentice Hall PTR

[B24] Widrow B Glover JJR McCool JM Kaunitz J Williams CS Hearn RH Zeidler JR Eugene JD Goodlin RC Adaptive noise cancelling: Principles and applications Proc IEEE 1975 63 12 1692 1716

[B25] Iannetti GD Niazy RK Wise RG Jezzard P Brooks JCW Zambreanu L Vennart W Matthews PM Tracey I Simultaneous recording of laser-evoked brain potentials and continuous, high-field functional magnetic resonance imaging in humans NeuroImage 2005 28 3 708 719 10.1016/j.neuroimage.2005.06.060 16112589

[B26] Ryali S Glover GH Chang C Menon V Development, validation, and comparison of ICA-based gradient artifact reduction algorithms for simultaneous EEG-spiral in/out and echo-planar fMRI recordings NeuroImage 2009 48 2 348 361 10.1016/j.neuroimage.2009.06.072 19580873 PMC2745974

[B27] Doblinger G MATLAB–Programmierung in der digitalen Signalverarbeitung 2001 Weil der Stadt: J. Schlembach Fachverlag

